# Advancing Inclusive Care: Embedding Health Equity in Palliative Care Fellowship Training

**DOI:** 10.1177/26892820251408397

**Published:** 2026-02-26

**Authors:** Sudha Natarajan, Alexis Drutchas, Carine Davila, Kyle Kozelka, Perla Macip Rodriguez, Alexandra Manyak, Kathleen Doyle, Jane deLima Thomas

**Affiliations:** 1 Division of Palliative Care and Geriatric Medicine, Massachusetts General Hospital, Boston, Massachusetts, USA.; 2 Department of Supportive Oncology, Dana Farber Cancer Institute, Boston, Massachusetts, USA.; 3 Department of Community Health, Tufts University, Medford, Massachusetts, USA.

**Keywords:** disparities, health equity curriculum, implicit bias, microaggressions, palliative care fellowship, structural racism

## Abstract

**Background:**

Providing quality palliative care requires an understanding of the causes of structural inequities that lead to disparities in health outcomes for minoritized patients.

**Methods:**

The Harvard Interprofessional Palliative Care Fellowship program (Boston, MA) is a year-long fellowship comprised of physicians, nurse practitioners, a social worker, and a pharmacist training in either adult or pediatric palliative care. A novel health equity curriculum, consisting of a day-long retreat, incorporation of health equity content into existing core didactics and quarterly health equity didactic sessions, was developed and integrated into the existing educational structure of the fellowship. The content included topics such as understanding implicit bias, structural inequities and trauma-informed care. The fellows’ perceptions of the curriculum were evaluated using qualitative analysis and retrospective pre-post survey methodology.

**Results:**

Sixteen fellows completed the health equity retreat evaluation and 18 fellows completed the end-of-year evaluation. The data demonstrated an increase in percentage of fellows reporting: ability to identify how structural racism affects the health of palliative care patients (44.4% to 94.4%) and comfort speaking up about racism and/or microaggressions (38.9% to 94.4%). Importantly, 88.9% of fellows reported that the learning environment felt safe and conducive to self-reflection and sharing.

**Conclusion:**

This novel health equity curriculum was effective in imparting health equity-related knowledge and skills in a safe learning environment.

## Key Message

This article describes the development and integration of a health equity curriculum into an interprofessional palliative care fellowship program. The results suggest that fellows developed skills to respond to racism/microaggressions and increased their understanding of issues related to health equity in palliative care.

## Introduction

Developing trusting, authentic relationships with patients, caregivers, and families is essential to providing quality palliative care. Disparities in access to quality palliative care and hospice services have been well documented.^1–6^ Most palliative care clinicians recognize that health inequities affecting historically marginalized communities erode trust in the health care system and negatively impact health outcomes.^7^ However, research shows that many clinicians do not feel equipped to address these impacts and lack the tools to improve trust between patient and provider.^8,9^

This study describes the development and integration of a novel health equity (HE) curriculum into a year-long interprofessional palliative care fellowship and assesses fellows’ perceptions of the impact of this curriculum. To our knowledge, this is the first report of such an effort, although many programs across the country are actively engaged in this work.

## Methods

### Core faculty development

The Harvard Interprofessional Palliative Care Fellowship Program is comprised of interprofessional faculty from Dana Farber Cancer Institute, Brigham and Women’s Hospital, and Massachusetts General Hospital. The HE core faculty and larger didactic faculty represent a breadth of identities and lived experiences, all of whom have experience or a deep interest in HE education. Several faculty have received additional graduate and fellowship training in public health and public policy, focusing on understanding disparities in care and improving care delivery for minoritized populations. In addition, members of our core and didactic faculty serve in leadership positions related to HE at their respective institutions. Faculty with more training and experience served as content experts for faculty who are newer in this work.

### Curriculum development

The American Academy of Hospice and Palliative Medicine’s (AAHPM) Entrustable Professional Activities and AAHPM’s Core Competencies were reviewed to identify competencies that specifically relate to HE.^10,11^ To ensure that HE content was integrated into the curriculum, rather than simply added on, all didactic faculty were asked to address HE issues relevant to their content area. Not all fellowship faculty have experience in HE education; thus, the core faculty were available as resources, and a publicly available checklist and video guide were provided for additional guidance.^12,13^ As an example of content integration, the pain management didactic was changed to include studies showing that cancer patients from minoritized populations were less likely to receive opioid therapy and included guidance about identifying potential bias in pain assessment.^1^

Additionally, a day-long HE retreat was added to the first week of the fellowship year. The morning included creation of community agreements to build the framework for a supportive and accountable learning environment, and a series of didactic sessions covering structural inequities in palliative care, implicit bias, upstander/microaggressions training, and the history of Boston’s changing racial demographics.^14^ In the afternoon, fellows participated in a National Park Service guided tour of Boston’s Black Heritage Trail, which provided insights into the historical factors that shape the communities served by the training institutions. Lastly, quarterly hour-long didactic sessions on microaggressions/upstander skills practice, intersectionality, and trauma-informed care, were created. Table 1 summarizes the curricular content presented during the orientation day and the quarterly didactic sessions, including the time devoted to each topic.

**Table 1.** Breakdown of Health Equity Curriculum, Including Time Dedicated to Each Topic

Health equity orientation day	
Didactic topic	Time spent in minutes
Community agreements	20
Structural inequities	60
Implicit bias	60
Upstander/Microaggressions training	60
History of Boston’s changing racial demographics	90
Black heritage trail tour	90
Quarterly Didactics	
Upstander/Microaggressions skills practice	60
Intersectionality	60
Trauma-informed care	60

### Evaluation

The curriculum was evaluated in academic years (AY) 2021 through 2023, i.e., July 2021 through June 2024. Since AY21 marked the pilot implementation of the HE retreat, informal feedback was gathered from fellows and faculty to inform curriculum development for the rest of the year. During AY21, the quarterly didactic sessions were evaluated by qualitative analysis, and these data were analyzed for common themes according to standard approaches to analysis of qualitative data.^15,16^ Two authors (S.N. and A.D.) independently reviewed responses to identify common themes. Consensus regarding themes was reached without the need for a third reviewer to resolve conflict.

Starting in AY22, evaluations were conducted using retrospective pre-post survey methodology and responses were gathered on a 5-point Likert Scale.^17^ All data were collected anonymously and gathered in aggregate. Given the limited power of our study, descriptive statistics were used to assess impact of the curriculum. The methodology was reviewed by Mass General Brigham’s Institutional Review Board (IRB), which determined the work did not fall into the category of human subjects research and was exempt from further IRB review.

### Statistical analysis

Likert Scale responses were recoded into binomial variables. For the orientation survey, “very good” and “excellent” were combined into one category, and “good,” “fair,” and “poor” were combined into a second category. For the year-end evaluations, “agree” and “strongly agree” were combined into one category, and “neutral,” “disagree,” and “strongly disagree” were combined into a second category. The McNemar’s exact test with continuity correction was used to assess statistical significance between paired responses in the pre-post surveys. A *p*-value less than 0.05 indicates statistical significance. Analyses were calculated in Excel.

## Results

Each Harvard Interprofessional Palliative Care Fellowship class is comprised of physicians trained in adult medicine, physicians trained in pediatric medicine, a pediatric social worker, adult and pediatric nurse practitioners, and a pharmacist. From AY21 through AY23, a total of 44 fellows received the HE curriculum. Demographic characteristics of the fellows are represented in Table 2.

**Table 2.** Demographics

Characteristics	*N* (%)
Gender	
Male	10 (27.7)
Female	34 (72.3)
Race	
White	28 (63.6)
Black or African American	6 (13.6)
Asian	3 (6.8)
Hispanic/Latino	5 (11.3)
Other	3 (6.8)
Chose not to identify	2 (4.5)
Profession	
Adult Physician	21 (47.7)
Pediatric Physician	7 (15.9)
Adult NP	7 (15.9)
Pediatric NP	3 (6.8)
Pediatric Social Work	3 (6.8)
Pharmacy	3 (6.8)

Note: Some fellows identified as more than one race. All those identities are included in this table, and as such, the percentages of racial identities do not sum to 100%.

Qualitative feedback was gathered for the quarterly HE sessions that addressed implicit bias, microaggressions/upstander skills practice, and trauma-informed care. Qualitative analysis demonstrated impact in two key domains: empowerment and empathy (Table 3). Empowerment was reflected through fellows developing skills to recognize their own implicit biases and to respond to microaggressions in their daily clinical practice. One fellow shared: “the exercise helped me understand that I have biases, even when I was ashamed to have them, it was good to share them with other people in the room to acknowledge them and start working on them.”

**Table 3.** AY21 Qualitative Analysis of Health Equity Didactic Sessions

Evaluation question	Theme	Representative quote
Implicit bias - Looking back before the implicit bias activity, how well did you understand that we each carry implicit biases and how these might affect our interactions with patients? How well do you feel you understand the role of implicit bias now?	Empowerment	“The exercise helped me understand that I have biases, even when I was ashamed to have them, it was good to share them with other people in the room to acknowledge them and start working on them. These biases definitely affect the way I interact with my patients and may affect our patient-physician relationship.”
Upstander training - Looking back to before the Upstander Training session, how well did you understand the definition of microaggressions and how to respond when you witness them? How equipped do you feel to respond to microaggressions now?	Empowerment	“Going through different scenarios and examples has helped me understand and identify when microaggressions happen and has provided me with tools to respond to them.”
Structural Inequities - Looking back to before the structural inequities didactic, how well did you understand how structural dynamics and barriers affect our patients’ experiences and the role of clinicians in combating these inequities? How well do you feel you understand structural inequities now?	Increased empathy	“The session broaden[ed] my understanding and helped me realize that I have to actually place myself in my patient’s shoes in order to understand how those inflicted structural dynamics and barriers have and will affect their care.”

Empathy was demonstrated through understanding that structural inequities unfairly disadvantage certain patient populations, negatively impacting their experiences with health care systems. One fellow shared: “the session broaden[ed] my understanding and helped me realize that I have to actually place myself in my patient’s shoes in order to understand how those inflicted structural dynamics and barriers have and will affect their care.”

### HE retreat survey results

For AY22–23, the orientation day pre-post survey was conducted on a 5-point Likert scale: poor, fair, good, very good, and excellent (Table 4). Sixteen of 29 fellows responded, yielding a response rate of 55.2%. The percentage of fellows who responded very good or excellent after the HE retreat increased for every question: understanding of my own implicit bias (25% to 56.3%); my fundamental understanding of concepts related to HE (31% to 75%); my understanding of the impact of structural inequities on patient health (50% to 81.3%); my ability to identify how structural racism affects the health of palliative care patients (18.8% to 50%), my level of comfort discussing the impact of structural inequities on patient health with colleagues (18.8% to 50%) and my level of comfort discussing the impact of structural inequities on patient health with colleagues I do not regularly work closely with (12.5% to 43.8%).

**Table 4.** Retrospective Pre-Post HE Retreat Evaluation, AY22 and AY23, *n* = 16

	Responded *very good* or *excellent*, no. (%)			
	Pre	Post	Point change, %	*p* value^a^
I have an understanding of my own implicit biases	4 (25.0)	9 (56.3)	+31.3	0.13
I have a fundamental understanding of concepts related to health equity	5 (31.3)	12 (75.0)	+43.8	0.02^b^
I have a fundamental understanding of concepts related to health equity in palliative care	1 (6.3)	8 (50.0)	+43.8	0.02^b^
I have a fundamental understanding of the impact of structural inequities on patient’s health	8 (50.0)	13 (81.3)	+31.3	0.07
I feel comfortable in discussing the impact of structural inequities on patients’ health with my colleagues	3 (18.8)	8 (50.0)	+31.3	0.07
I feel comfortable discussing health equity issues with colleagues I do not regularly work closely with	2 (12.5)	7 (43.8)	+31.3	0.13
I can identify how structural racism affects the health of my palliative care patients	5 (31.3)	10 (62.5)	+31.3	0.02^b^

a Calculated using McNemar’s exact test for paired, binary data, using continuity correction for discrete distribution.

b *p* < 0.05 indicates statistical significance.

### Year-end evaluation results

Year-end evaluations were also conducted on a 5-point Likert scale: strongly agree, agree, neutral, disagree and strongly disagree (Table 5). The response rate for AY21–AY23 was 18/44 (40.9%). The percentage of fellows who answered agree or strongly agree increased for all questions: I have a fundamental understanding of concepts related to HE (61.1% to 100%); I have a fundamental understanding of concepts related to HE in palliative care (27.8% to 94.4%); I can identify how structural racism affects the health of palliative care patients (44.4% to 94.4%), I can identify instances of racism and/or microaggressions in clinical practice (38.9% to 94.4%); I feel comfortable speaking up about racism and/or microaggressions (38.9% to 94.4%). Importantly, 88.9% of fellows responded either *agree* or *strongly agree* when asked whether the learning environment felt safe and conducive to self-reflection and sharing with the group.

**Table 5.** Retrospective Pre-Post End-of-Year Evaluation, AY21 through AY23, *n* = 18

	Responded *agree* or *strongly agree,* number (%)			
	Pre	Post	Point change, %	*p* value^a^
I have a fundamental understanding of concepts related to health equity.	11 (61.1)	18 (100.0)	+38.9	0.02^b^
I have a fundamental understanding of concepts related to health equity in palliative care.	5 (27.8)	17 (94.4)	+66.7	0.001^b^
I can identify instances of racism and/or microaggressions in my clinical practice that affect colleagues and/or patients.	9 (50.0)	18 (100.0)	+50.0	0.003^b^
I feel comfortable speaking up about racism and/or microaggressions when I encounter them in my clinical practice.	7 (38.9)	17 (94.4)	+55.6	0.003^b^
I can now identify how structural racism affects the health of palliative care patients.	8 (44.4)	17 (94.4)	+50.0	0.004^b^

a Calculated using McNemar’s exact test for paired, binary data, using continuity correction for discrete distribution.

b *p* < 0.05 indicates statistical significance.

## Discussion

Over recent years, institutions for medical education have begun to incorporate HE education into their curricula. A 2022 study examining the outcomes of a HE curriculum in a family medicine residency program found that the curriculum successfully increased residents’ ability to care for vulnerable patients.^8^ A 2019 study demonstrated that a curriculum focused on HE, diversity, and inclusion helped pediatric residents become better advocates for their patients.^18^ To our knowledge, this is the first report describing the integration of a HE curriculum into a palliative care postgraduate fellowship program.

Health inequities and structural racism can be difficult to discuss openly and are prone to misunderstandings. To create an accountable and nurturing learning environment, fellows were empowered to develop community agreements at the beginning of the HE retreat, which served as mutually agreed-upon expectations for how each fellow would engage with this work. Principles of these community agreements included, engaging with curiosity and avoiding assumptions about the intent of another’s words and actions, and separating the intent of one’s words and actions from the impact on another, allowing space for open dialogue and repair if necessary. Creation of a learning environment in which fellows felt comfortable participating was fundamental to the success of this curriculum.

This report has several limitations. While our results suggest an overall positive impact among fellows, the Likert scales used for the post-orientation and end-of-year surveys are different. This does not affect interpretation of the results themselves; however, it is more difficult to draw conclusions about the overall impact of the curriculum. The difference in the Likert scales is a consequence of design choices made during the initial development of the evaluation instruments. All future evaluation surveys are being conducted with consistent Likert scales. Further, higher response rates would improve our ability to apply more robust statistical methods for analysis. Lastly, given time constraints of a year-long fellowship, we were challenged to be very selective in deciding which HE content was essential to incorporate.

Initial evaluation of this novel HE curriculum suggests that fellows gained knowledge and skills to identify health disparities in palliative care and respond effectively to racism/microaggressions in a safe learning environment. Further evaluation is needed to determine whether this curriculum leads to changes in clinical practice or sustained engagement with HE issues beyond the fellowship year. We acknowledge that other institutions and palliative care fellowship programs are also developing similar curricula, and we hope our experience contributes to the ongoing dialogue about best practices for incorporating this important educational content into palliative care training.

### Future directions

Our hope is that exposure to HE education in palliative care training imparts skills that fellows integrate into their future careers. An important next step would be to evaluate how prior fellows are engaging in HE work beyond fellowship and whether they are applying the knowledge gained from the above curricular additions. Further, we recognize that year after year, learners enter fellowship with an increasingly sophisticated knowledge about HE issues in palliative care. It is important that we continue to evolve the curriculum to meet the needs of our learners. To this end, soliciting yearly feedback about content that was novel versus content that was redundant from prior training is essential.

## Authors’ Contributions

S.N.: Conceptualizing (supporting), data curation (equal), formal analysis (lead), methodology (supporting), visualization (lead), writing original draft (equal), and writing—review and editing (lead). A.D.: Conceptualizing (supporting), data curation (equal), methodology (equal), writing original draft (equal), and writing—review and editing (supporting). C.D.: Conceptualizing (supporting), methodology (equal), and writing—review and editing (supporting). K.K.: Conceptualizing (supporting), methodology (equal), and writing—review and editing (supporting). P.M.R.: Conceptualizing (supporting) and writing—review and editing (supporting). A.M.: Writing—original draft (supporting) and writing—review and editing (supporting). K.D.: Conceptualizing (equal), supervision (equal), and writing—review and editing (supporting). J.D.T: Conceptualizing (equal), supervision (equal), and writing—review and editing (supporting).

## Abbreviations

AAHPM: American Academy of Hospice and Palliative Medicine
AY: Academic Year
HE: Health Equity
IRB: Institutional Review Board
